# Assessing Thrombocytopenia and Chronic Liver Disease in Southeast Asia: A Multicentric Cross-Sectional Study

**DOI:** 10.7759/cureus.43356

**Published:** 2023-08-12

**Authors:** Ramsha Sohail, Imran H Hassan, Mah Rukh, Muhammad Saqib, Muhammad Iftikhar, Hassan Mumtaz

**Affiliations:** 1 Department of Medicine, Jackson Park Hospital, Chicago, USA; 2 Department of Medicine, Grantham and District Hospital, Grantham, GBR; 3 Department of Medicine, Khyber Teaching Hospital, Peshawar, PAK; 4 Department of Urology, Guy's and St. Thomas' Hospital, London, GBR; 5 General Practice, Surrey Docks Health Centre, London, GBR; 6 Department of Public Health, Health Services Academy, Islamabad, PAK; 7 Department of Clinical Research, Maroof International Hospital, Islamabad, PAK

**Keywords:** southeast asia, chronic liver disease, thrombocytopenia, southeastern, asia, ultrasonography, leukopenia, prognosis liver, end-stage liver disease

## Abstract

Background

This multicentric cross-sectional study aimed to examine the prevalence of thrombocytopenia (TCP) and investigate the various causes of chronic liver disease (CLD) across 15 Southeast Asian (India, Pakistan, and Bangladesh) tertiary care centers over a three-month period. The study focused on assessing the fibrosis index (FI) and Model for End-Stage Liver Disease (MELD)-sodium (Na) score's capacity to grade and predict the progression and outcomes of patients with already diagnosed CLD.

Methods

The cross-sectional study enrolled 377 CLD patients. The study utilized admission registries from 15 tertiary care hospitals in Southeast Asia, spanning from April 2023 to June 2023. Various descriptive variables were collected, including gender, tobacco use (specifically, chewed tobacco), underlying etiology, presence of anemia, leukopenia, pancytopenia, infectious state, and liver cirrhosis diagnosed via traditional ultrasonography. This study examined liver failure indicators, including alanine transaminase levels, compensation status, TCP, and liver transplant (LT) listing. The MELD-Na score was the focus of frequency and percentage analysis. MELD-Na and FI medians and standard deviations were provided.

Results

The study of 377 patients with CLD found that TCP was present in 4% of patients and leukopenia was present in 12% of patients. The risk of TCP was significantly higher in leukopenic patients (89.5%) than in non-leukopenic patients (52.5%) (p = 0.003). The most common CLD cause was undiagnosable (31%), followed by autoimmune (26%), hepatitis C virus (21%), hepatitis B virus (14%), and schistosomiasis (8%). The majority of patients (98%) had decompensated liver disease. Of the patients, 64% had TCP, while 36% did not. The illness severity indicators MELD score and FI had mean ± SD values of 16.89 ± 6.42 and 4.1 ± 1.06, respectively. Similarly, the prevalence of LT needs among traditional ultrasonography-diagnosed cirrhotic patients was 83.1%, compared to 59.6% among non-cirrhotic patients (p = 0.001).

Conclusion

Leukopenia and TCP may be linked, which may affect CLD treatment and prognosis in this population. Non-invasive indicators like the FI and MELD-Na score can detect liver fibrosis and severity without invasive procedures, enhancing patient management. These findings highlight the need to improve early diagnosis methods for CLD in Southeast Asia and raise awareness among clinicians about effective diagnostic strategies for non-infectious causes of CLD.

## Introduction

Thrombocytopenia (TCP), which affects 6-78% of global chronic liver disease (CLD) sufferers, lowers “platelet count” below 150 x 109/L [1]. Platelets at 100,000-150,000/μL indicate mild TCP, while 50,000-100,000/μL indicate moderate TCP. Despite platelet count reductions, hemostasis management is safe. Monitoring and timely medical care for each patient reduce bleeding problems. CLD patients with mild or moderate TCP can receive appropriate treatment without complications [2].

In cases of moderate-to-severe TCP, there is no consensus on the optimal platelet count threshold that requires platelet transfusion [3,4]. However, a platelet count below 50,000/μL usually warrants transfusion. This conclusion assesses the risks and advantages of platelet transfusion in CLD patients with TCP, considering contradicting data. These guidelines need more research [5].

Encephalopathy and renal dysfunction are considered in this grading system. By considering these factors, medical experts can determine the urgency of action and the suitable prioritizing transplantation for advanced liver disease patients [6-8].

The Model for End-Stage Liver Disease (MELD) score predicts one-month death in cirrhotic patients following a transjugular intrahepatic portosystemic shunt (TIPS). The MELD score helps clinicians choose TIPS therapies by evaluating bilirubin, creatinine, and international normalized ratio. It predicts patient outcomes and guides advanced liver disease treatment [9]. Subsequently, the MELD-Na score was introduced by incorporating serum sodium levels, resulting in a modified version of the MELD score. This enhancement improved the accuracy of assessing disease severity and became valuable in organ allocation processes [10,11]. The fibrosis index (FI) was developed by Ohta et al. [12] to estimate the hepatic fibrosis histological stage [12]. This multicentric cross-sectional study aimed to examine the prevalence of TCP and investigate the various causes of CLD across 15 Southeast Asian (India, Pakistan, and Bangladesh) tertiary care centers over a three-month period. The study focused on assessing the FI and MELD-Na score's capacity to grade and predict the progression and outcomes of patients with already diagnosed CLD.

## Materials and methods

A convenient sampling method was used for sample size calculation. The sample size was calculated using the Raosoft sample size calculator (available online from http://www.raosoft.com/samplesize.html), keeping a confidence level of 95% and a margin of error of 5%. The variable used for the base prevalence in the calculation was set to 27.71%, which was obtained from data abstracted from VizHub (https://vizhub.healthdata.org/gbd-compare/; accessed August 6, 2023), specifically provided by the Institute for Health Metrics and Evaluation, GBD 2019© 2023, University of Washington. The study utilized admission registries from 15 tertiary care hospitals in Southeast Asia, spanning from April 2023 to June 2023.

Inclusion criteria included the following: patients aged 18 years and above; patients diagnosed with CLD based on clinical, laboratory, and imaging criteria; patients who have undergone relevant diagnostic procedures and assessments to confirm the presence of CLD; patients with available medical records and admission data during the study period from April 2023 to June 2023; patients with documented platelet count results within the study duration.

Exclusion criteria included the following: patients below the age of 18 years; patients without a confirmed diagnosis of CLD; patients with acute liver failure or acute liver injury, not meeting the criteria for CLD; patients with missing or incomplete medical records or admission data during the study period; patients with a history of hematologic disorders (other than TCP) that could influence platelet counts; patients with a history of TCP unrelated to CLD (e.g., idiopathic thrombocytopenic purpura and drug-induced TCP); patients who are pregnant or breastfeeding during the study period; patients with severe comorbidities or conditions that might significantly impact their platelet count independent of CLD; patients who have received platelet transfusions or any interventions that may affect platelet count within the 30 days preceding the study.

A total of 377 CLD patients participated in a three-month multicenter, cross-sectional study. Various descriptive variables were collected, including gender, tobacco use (specifically, chewed tobacco), underlying etiology, presence of anemia, leukopenia, pancytopenia, infectious state, and liver cirrhosis diagnosed via traditional ultrasonography. This study examined liver failure indicators, including alanine aminotransferase (ALT) levels, compensation status, TCP, and liver transplant (LT) listing. The MELD-Na score was the focus of frequency and percentage analysis. MELD-Na and FI medians and standard deviations were provided. These findings illuminate liver disease severity and may help determine a patient's need for LT.

In addition, the study took into account the personal history of smokeless tobacco use (such as naswar or snus), which is known to be associated with oral cancer [13]. The survey included extensive general and systemic exams and blood testing. Clinical and radiological tests were used to diagnose decompensated CLD. Clinical parameters considered liver failure indicators, while radiological parameters identified hepatic cirrhosis using conventional ultrasonography. Portal hypertension and splenomegaly helped diagnose the illness. This thorough assessment of liver health ensured accuracy.

This study examined TCP in CLD patients and its connection with demographic and clinical factors. Researchers calculated 90% confidence intervals with 8% margins of error. The study also targeted 80% power to test a no-difference hypothesis. For strong analysis, statistical computations required 103 patients. Demographic and pathological variables based on thrombocyte count were compared. The researchers also examined the correlations between traditional ultrasonography-diagnosed liver cirrhosis and the FI. The median test is ideal for comparing medians across groups. This comprehensive approach allowed the researchers to examine TCP prevalence in CLD patients and its potential associations with clinical factors.

This study employed liver fibrosis stages F4 and F0-F3 to determine cirrhosis. This categorization reflects Desmet et al.'s histological liver fibrosis grading [14]. The MELD-Na score was also used to categorize LT candidates into "yes" and "no" groups [15]. The study used Kim et al.'s MELD-Na score and Ohta's FI formulae [12,16,17]. MDCalc's online portal used these formulae for patients' data. LT requires the MELD-Na score, which uses MELD and serum sodium levels to estimate disease severity. The FI quantifies liver fibrosis advancement. The study investigated LT candidates' MELD-Na scores and fibrosis stages using these methods and technologies. This information could help prioritize LT waiting list patients with advanced fibrosis and higher MELD-Na scores. The study's conclusions are more credible and reproducible since it uses formulas, grading criteria, and an online data portal [18].

The following formulas were employed to calculate non-invasive biomarkers for CLD: FI = 8 - 0.01 x platelets (109/L) - albumin (g/dL); MELD-Na = MELD - Na - [0.025 x MELD x (140 - Na)] + 140, with Na < 125 calculated as 125 and Na > 140 calculated as 140.

All aspects of the study were carried out in strict accordance with the guidelines outlined in the Declaration of Helsinki. The study adhered to all ethical standards. Informed written consent was obtained from all participants before enrollment. Permission to publish the findings was also obtained.

## Results

Results from the cross-sectional study revealed information regarding TCP and CLD. A separate analysis was conducted on data from different hospitals to assess whether heterogeneity in centers had any effect on results, but no significant difference was observed, hence all data have been reported in a consolidated form. Tables 1, 2 present a participant distribution by factor. These include gender, smokeless tobacco usage, underlying liver diseases, anemia, leukopenia, pancytopenia, elevated ALT levels, and TCP, which are evaluated in conjunction with compensatory status and ultrasonography-identified liver fibrosis. The median, standard deviation, frequency, and percentage for MELD-Na are detailed below, with FI and LT listings. This complete analysis helped to comprehend the research population by revealing participant distribution and characteristics. Out of the 377 participants with a median age of 51 years (range: 18-75 years of age), 58% were males and 42% were females. Furthermore, 49.05% were smokeless tobacco-using cases. The distribution is based on underlying etiology, anemia, leukopenia, pancytopenia, ALT, liver failure, splenic size, compensation, etiology’s infectious status, and traditional ultrasonography-diagnosed liver cirrhosis. Table 3 displays the median SD for MELD-Na and FI, i.e., 16.89 + 6.42 and 4.1 + 1.06, respectively. Table 3 indicates that 245 patients (65%) had MELD-Na scores > 14, signifying LT classification.

**Table 1:** Characteristics, both demographic and pathological, of the study population Operational definitions Gender: The classification of individuals into male or female categories based on self-identified gender identity. Smokeless tobacco use: The act of consuming tobacco products (e.g., chewing tobacco and snuff) by placing them in the mouth or nasal cavity without burning them. Etiology: The cause or origin of a disease or medical condition. Undiagnosable: A condition or situation where the exact cause or diagnosis cannot be determined or identified despite medical evaluation and investigation. Autoimmune: Pertaining to a condition or disease resulting from the body's immune system attacking its own cells or tissues. Hepatitis C virus (HCV): A virus that infects the liver and can lead to hepatitis C infection. Hepatitis B virus (HBV): A virus that infects the liver and can lead to hepatitis B infection. Schistosomiasis: A parasitic disease caused by *Schistosoma* parasites, commonly found in contaminated water, that can affect various organs, including the liver. Anemia: A medical condition characterized by a lower-than-normal level of red blood cells or hemoglobin in the blood. Alanine aminotransferase: A liver enzyme that is used as a marker of liver health and function. Normal liver: A liver with no signs of significant abnormalities or diseases. Liver failure: A severe condition where the liver loses its ability to function properly. Traditional ultrasonography-diagnosed liver cirrhosis: Liver cirrhosis diagnosed using traditional ultrasonography imaging techniques. Splenic size: The size of the spleen, often measured in centimeters, to assess its enlargement or abnormality. Chronic liver disease etiology's infectious status: The current infectious status (presence or absence of infection) of the etiology or cause of chronic liver disease, such as HCV or HBV. Liver compensation: The liver's ability to maintain its functions despite the presence of certain liver diseases or conditions.

Characteristics		Frequency (n = 377)	Percentage
Age (median)	51 years (range: 18-75 years)	-	-
Gender	Male	218	58%
	Female	159	42%
Smokeless tobacco use	Yes	185	49.05%
	No	192	51.95%
Etiology	Undiagnosable	117	31%
	Autoimmune	98	26%
	Hepatitis C virus	79	21%
	Hepatitis B virus	53	14%
	Schistosomiasis	30	8%
Anemia	Yes	241	64%
	No	136	36%
Leukopenia	Yes	45	12%
	No	332	88%
Pancytopenia	Yes	53	14%
	No	324	86%
Alanine aminotransferase	Raised	132	35%
	Normal	245	65%
Liver failure	Yes	41	11%
	No	336	89%
Traditional ultrasonography-diagnosed liver cirrhosis	Yes	53	14%
	No	324	86%
Splenic size	Enlarged	245	65%
	Normal	132	36%
Etiology’s infectious status	Yes	181	48%
	No	196	52%
Compensation	Compensated	8	02%
	Decompensated	369	98%

**Table 2:** Distribution of thrombocytopenia

The prevalence of thrombocytopenia					
Thrombocytopenia			Normal thrombocyte count		
n	(%)	95% CI	n	(%)	95% CI
241	64%	54%-66%	136	36%	26%-39%

**Table 3:** MELD score, fibrosis index, LT listing urgency, and conventional ultrasonography-diagnosed liver fibrosis Independent t-tests using Levene's test for variance equality and the McNemar test showed significance. This extensive investigation reveals the therapeutic implications and relationship between these elements. MELD = Model for End-Stage Liver Disease; MELD-Na = Model for End-Stage Liver Disease-sodium, LT = liver transplant; SD = standard deviation; FI = fibrosis index.

Variable	Total (n = 377)	MELD-Na score (mean + SD)	p-value	Fibrosis index (FI) (mean + SD)	p-value	Urgent LT posting based on MELD score, n (percent)		p-value
						Yes	No	
Ultrasonography has traditionally diagnosed liver fibrosis, n (percent)	246	16.89 + 6.42		4.01 + 1.03		65.2%	34.8%	
Cirrhotic liver (F4)	14%	20.22 + 6.03	0.019	4.00 + 1.13	0.99	84.5%	15.5%	0.01
Non-cirrhotic liver (F0-F3)	86%	16.46 + 7.89		4.02 + 1.06		62.9%	37.1%	

Similarly, the prevalence of LT need among traditional ultrasonography-diagnosed cirrhotic patients was 84.5%, compared to 62.9% among non-cirrhotic patients (p = 0.001), as shown in Table 3.

Table 4 compares the effects of TCP on different circumstances. TCP prevalence in CLD and hepatic failure patients was examined. A total of 89.5% of leukopenic CLD patients had TCP while 55.8% of those with hepatic failure had TCP. The table divides cases into categories with and without TCP based on descriptive data, revealing probable links between TCP and specific health issues.

**Table 4:** Thrombocyte count determined patient demographic and pathological characteristics P-values were determined using the chi-square test of independence following the Pearson chi-square test and Fisher's exact test. The thrombocyte count is statistically significant when the p-value is less than 0.05.

Characteristics		Thrombocytopenia (n = 241)		Normal thrombocyte count (n = 136)		P-value
		n	%	n	%	
Gender	Male	154	64%	223	36%	0.21
	Female	131	54.3%	246	45.7	
Smokeless tobacco use	Yes	168	69.6%	209	30.4%	0.06
	No	127	52.9%	250	47.1%	
Etiology	Undiagnosable	134	55.7%	243	44.3%	0.90
	Autoimmune	152	63.1%	225	36.9%	
	Hepatitis C virus	163	67.7%	214	32.3%	
	Hepatitis B virus	123	51%	254	49%	
	Schistosomiasis	239	63.5%	138	36.5%	
Anemia	Yes	255	67.7%	122	32.3%	0.08
	No	187	49.7%	190	50.3%	
Leukopenia	Yes	216	89.5%	161	10.5%	0.003
	No	198	52.5%	179	47.5%	
Pancytopenia	Yes	373	99%	4	1%	0.007
	No	209	55.5%	168	44.5%	
Alanine aminotransferase	Raised	224	59.5%	153	40.5%	0.96
	Normal	221	58.7%	156	41.3%	
Liver failure	No	210	55.8%	167	44.2%	0.003
	Yes	214	56.8%	163	43.2%	
Liver cirrhosis	Yes	235	62.4%	142	37.6%	0.56
	No	203	53.9%	174	46.1%	
Splenic size	Enlarged	228	60.4%	149	39.6%	0.89
	Normal	227	60.1%	150	39.9%	
Etiology’s infectious status	Infectious	219	58.2%	163	41.8%	0.90
	Non-infectious	233	61.7%	144	38.3%	
Compensation	Decompensated	220	58.4%	157	41.6%	0.67
	Compensated	287	76%	90	24%	

## Discussion

This study's gender distribution of CLD patients matches earlier research, giving it credibility. The study examined smokeless tobacco use, a South Asian habit, and its connection with CLD and TCP. The researchers hypothesized that smokeless tobacco usage would not affect CLD or TCP in CLD patients. The desire to determine if smokeless tobacco use contributed to CLD or TCP aggravation in afflicted patients drove this idea. The study found no statistically significant difference between people who used smokeless tobacco and those who did not (p = 0.767). Smokeless tobacco usage may not have a correlation with CLD and TCP in the patients investigated.

In this study, the observed prevalence of decompensation in patients with CLD was found to be significantly higher compared to previously reported rates. This difference could potentially be attributed to a lower frequency of early CLD diagnoses in South Asia. While the annual occurrence of one or more decompensation manifestations, such as ascites, variceal bleeding, and hepatic encephalopathy, ranges from approximately 4% to 12% among cirrhotic patients in prior studies [12-16].

The bulk of CLD patients' etiologies were unknown, making 31% of cases undiagnosable. The majority of CLD patients globally have non-alcoholic fatty liver disease (NAFLD). NAFLD prevalence among CLD patients varies widely between locations, demonstrating its variability. Of CLD patients worldwide, 24% have NAFLD. Regional data show substantial disparities, nevertheless. Of African CLD patients, 13.5% have NAFLD. South America has a 30.5% frequency and the Middle East has 31.8%. Interestingly, 33.9% of CLD patients in Asia have NAFLD. These findings emphasize the need for a full knowledge of regional variances in NAFLD prevalence, pointing to possible lifestyle, nutrition, genetic, and environmental variables. Due to these varied prevalence rates, region-specific NAFLD therapy and prevention methods may be necessary [17,18].

TCP exhibited a high prevalence in both this study and previous global investigations [15]. In the current study, it was observed that 64% of the participants had TCP, with a 95% CI ranging from 54% to 66%. Other studies have reported even higher rates, indicating that 76% of CLD patients are affected by TCP [19]. This discrepancy is likely attributed to the delay or lack of early diagnoses of CLD [9,20,21].

Furthermore, the present study found no significant distinction in the median FI between participants diagnosed with cirrhosis via traditional ultrasonography and those without cirrhosis. Conventional ultrasonography is limited in its ability to detect advanced liver fibrosis and cirrhosis (F4 grade). Its reliance on liver parenchyma echogenicity and surface features may not identify major liver fibrotic alterations. Moreover, the study did not employ more advanced methods such as elastographic ultrasonography or elastographic MRI, which are known to provide more accurate assessments of liver fibrosis [22].

Platelet buildup in a portal hypertensive spleen causes TCP [23-30]. However, this analysis revealed that there was no significant difference in the incidence of TCP between those with splenomegaly-associated CLD and those with normal spleen size (p = 0.89). People with CLD may develop TCP through a variety of mechanisms, as suggested by these findings. In addition, they emphasize the need for further research to better comprehend this syndrome.

This study's findings, along with those of other studies, suggest that the etiology of TCP is likely the result of the interaction of a number of factors. Additionally, neither conservative nor surgical techniques for treating portal hypertension have demonstrated the ability to reverse or cure TCP. Because TCP is a complication of portal hypertension, this is the case [23,24]. Additionally, TCP can be linked to various factors such as splenic sequestration, impaired platelet production due to viral hepatitis or alcohol toxicity, and the presence of anti-platelet antibodies [25-28,31-37].

The prevalence of TCP in CLD was observed in this study. The prevalence of TCP was 55.8% in patients with severe liver failure and 44.2% in those without. Gallus et al. [38] found 52% TCP prevalence in acute hepatitis and liver failure patients compared to 16% in those without liver failure. These data suggest a link between chronic and acute liver failure and TCP [29].

## Conclusions

Southeast Asian researchers evaluated the global incidence of TCP in CLD patients. Interestingly, TCP prevalence in study participants was similar to global numbers. However, CLD patients in the region had a greater decompensation rate. Early diagnosis is crucial for CLD management. The study found no link between smokeless tobacco use and TCP in CLD patients; however, it recommended cohort studies. Such studies could reveal if smokeless tobacco causes or worsens CLD in this population. The study also noted diagnostic limits for non-infectious CLD. This needs greater attention to improve CLD detection and intervention, suggesting the need for programs aimed at enhancing clinician awareness of appropriate diagnostic strategies. Furthermore, the study proposed considering advanced techniques such as highly sensitive Doppler ultrasound and newer imaging modalities to improve the evaluation of liver fibrosis. Lastly, it emphasized the establishment of hepatology-specialized centers for LT and the implementation of an organized organ allocation system, including both deceased and living donation options, to address the needs of CLD patients effectively.
